# The Use of Drugs

**Published:** 1891-02

**Authors:** 


### THE USE OF DRUGS.

It is an inborn idea of the English and American mind that there must perforce be some drug adapted to the ever varying states of the body which contains it. So many disorders, so many remedies. The faith which never wavers that Nature has a panacea for every ill is touching to witness, and seems to flourish in an age of science and scepticism as vigorously as in the past. Americans especially exhibit a robust form of this faith. American girls, it is said, now carry about with them ornamental cut-glass bottles containing quinine pills, with which they dose themselves from time to time. If fatigued they take two pills ; if chilly, one ; if hungry (as they generally seem to be), four or five. It is said that ten is the correct dose for wet feet! We believe the misuse of drugs in America is inducing a lot of sensible people in that country to give them up altogether. That is the only safe way with drugs.—*Farm and Home, England.*

